# Hypochloremia: A Potential Indicator of Poor Outcomes in COVID-19

**DOI:** 10.3390/medicina60091414

**Published:** 2024-08-29

**Authors:** Orçun Barkay, Faruk Karakeçili

**Affiliations:** Department of Infectious Diseases and Clinical Microbiology, Faculty of Medicine, Erzincan Binali Yıldırım University, Erzincan 24100, Turkey; drfarukkarakecili@hotmail.com

**Keywords:** biomarker, COVID-19, D-dimer, hypochloremia, ICU, tocilizumab

## Abstract

*Background: Coronavirus* Disease-2019 (COVID-19) has posed formidable challenges to healthcare systems. Exploring novel biomarkers that can provide valuable prognostic insights, particularly in critically ill patients, has a significant importance. Against this backdrop, our study aims to elucidate the associations between serum chloride levels and clinical outcomes. *Methods:* A total of 499 patients were enrolled into the study. The serum chloride levels of patients upon hospital admission were recorded and then categorized into three groups (hypochloremia, normochloremia, and hyperchloremia) for the evaluation of clinical outcomes. Additionally, serum C-reactive protein, procalcitonin, and D-dimer measurements were recorded for further evaluation. *Results:* A total of 390 (78.1%) patients tested positive for COVID-19 via polymerase chain reaction testing. Non-contrast thorax computed tomography scans were indicative of COVID-19 compatibility for all patients. A total of 210 (42%) patients were female and 289 (58%) were male. A total of 214 (42.8%) patients necessitated tocilizumab intervention; 250 (50.1%) were at an intensive care unit (ICU), with 166 (66.4%) of them receiving tocilizumab. A total of 65 (13%) patients died, 40 (61.5%) of whom received tocilizumab; 41 (63%) were in the ICU. Serum chloride levels upon admission were markedly lower and elevated D-dimer levels were apparent in tocilizumab users, patients requiring ICU care, and patients who died. *Conclusions:* our findings provide robust evidence supporting the value of serum chloride levels as a prognostic biomarker in critically ill COVID-19 patients.

## 1. Introduction

*Coronavirus* Disease-2019 (COVID-19) has posed formidable challenges to healthcare systems globally [1,2,3]. Critical illness due to COVID-19 often involves a dysregulated immune response, leading to conditions such as a cytokine storm, acute respiratory distress syndrome (ARDS), and multiorgan dysfunction [4,5,6]. In this ongoing battle against the COVID-19 pandemic, there has been a continuous effort to decipher the complex interplay between clinical parameters and patient outcomes. Recent research underscores the significance of exploring novel biomarkers that can provide valuable prognostic insights, particularly in critically ill patients [7]. Therefore, identifying reliable predictors of disease severity and patient outcomes is of paramount importance.

Against this backdrop, our study aims to elucidate the associations between serum chloride levels (a proven parameter for a bad course of sepsis) upon hospital admission and critical clinical outcomes. By investigating the potential of hypochloremia as a prognostic biomarker, we aspire to contribute to a deeper understanding of COVID-19 pathophysiology and enhance the predictive accuracy of clinical models for disease severity and patient outcomes.

## 2. Materials and Methods

**Study Design and Setting:** This retrospective study was conducted at the Department of Infectious Diseases and Clinical Microbiology, Erzincan University Faculty of Medicine, Erzincan, Turkey. A total of 499 patients with a confirmed COVID-19 diagnosis by polymerase chain reaction enrolled in the study. The serum chloride levels of patients upon hospital admission were recorded for independent evaluation and then categorized into three groups: ≤97 mmol/L for hypochloremia, 98–107 mmol/L for normochloremia, and ≥108 mmol/L for hyperchloremia. Patients were excluded from the study if they had complaints such as vomiting or diarrhea, a history of diuretic use, severe lipemia, or signs of chronic respiratory acidosis, as these conditions can cause hypochloremia. Additionally, patients with a history of acetazolamide use, severe dehydration, or renal failure, which can cause hyperchloremia, were also excluded. Additionally, serum C-reactive protein (CRP), procalcitonin, and D-dimer measurements were recorded, and their associations with demographic data, serum chloride levels at admission, serum chloride level groups, and other parameters were scrutinized.

**Data Collection:** Medical records of the patients admitted to the hospital with confirmed COVID-19 were included in the study. A total of 53 patients were excluded due to reasons of possible hyperchloremia or hypochloremia. Patient demographic data, laboratory results, clinical outcomes, and treatment interventions were extracted from the hospital data processing system.

**Statistical Analysis:** Descriptive statistical analyses were conducted based on the nature and distribution of the variables under consideration. The normality of the observed parameters was assessed using a Kolmogorov–Smirnov test, revealing a departure from normal distribution. Correlation among numerical measurements was evaluated utilizing Spearman’s rank correlation analysis.

Associations between numeric serum chloride values and categorical variables, including gender, frequency of tocilizumab usage, instances of intensive care unit hospitalization, and mortality rate, were appraised using a Mann–Whitney U test. Receiver Operating Characteristic (ROC) analysis was employed to identify optimal cutoff values and gauge diagnostic efficacy. ROC analysis is a statistical method used to determine the diagnostic success of a test (highest specificity and sensitivity values), to compare the sensitivity and specificity values obtained at different cutoff points, to determine the specificity values corresponding to certain sensitivity values or the sensitivity values corresponding to certain specificity values, and to compare the diagnostic success of two or more tests.

The connection between serum chloride level groups and numerical measurements was investigated via a Kruskal–Wallis test, while the interplay between categorical variables was examined using a Pearson chi-square test. Statistical significance was acknowledged at a threshold of *p* ≤ 0.05, and a statistical software package (SPSS ver. 23) was employed for all calculations.

**Ethical approval:** Study approval was obtained from the scientific studies program of the Turkish Ministry of Health with the approval number 2021-01-27T15_09_55. Also, ethical approval was gained from the Ethics Committee of Erzincan Binali Yıldırım University (Number: 2023-22/8; Date: 14 December 2023). Informed consent was not obtained because of the retrospective design of the study.

## 3. Results

Among 499 patients, 390 (78.1%) patients tested positive for COVID-19 via polymerase chain reaction testing. Non-contrast thorax computed tomography scans were indicative of COVID-19 compatibility for all patients. Among the participants, 210 (42%) were female and 289 (58%) were male. The mean age was 61.9 ± 12.4 years, ranging from 26 to 95. During their course of observation, 214 (42.8%) patients necessitated tocilizumab intervention. Within the cohort, 250 (50.1%) patients were under intensive care unit monitoring, with 166 (66.4%) of them receiving tocilizumab treatment. A total of 65 (13%) people died, 40 (61.5%) of whom received tocilizumab treatment; 41 (63%) were monitored in the intensive care unit.

Directly considering serum chloride measurements upon admission, there were no substantial alterations in the serum chloride level, CRP, procalcitonin, D-dimer, and total length of hospital stay associated with age. The serum chloride level upon admission exhibited a modest yet statistically significant inverse correlation solely with D-dimer, while no significant links were established with CRP, procalcitonin, and total hospital stay.

Evaluation of the descriptive statistics of measurements categorized by gender showed the serum chloride level at admission had been slightly elevated in women, reaching statistical significance. However, the observed mean variance lacks clinical significance. Other measurements exhibited no significant gender-based distinctions.

Individuals employing tocilizumab displayed a significantly higher mean age by approximately 3.8 years. The serum chloride level upon admission was markedly lower, and elevated D-dimer levels were apparent in tocilizumab users. Prolonged total hospital stays were also observed among these patients. Descriptive statistics of measurements contingent on tocilizumab utilization are presented in Table 1.

Employing a serum chloride level threshold of 101.5 upon admission, individuals surpassing this value were projected to evade the cytokine storm, yielding a diagnostic success rate of 64.2% and a prediction success rate of 58.9% for identifying those prone to the cytokine storm.

With a D-dimer threshold of 933.5, individuals exceeding this value were considered susceptible to the cytokine storm, achieving diagnostic success at 70.6% and predicting those resistant to the cytokine storm at 60.7%.

The descriptive statistics pertaining to measurements in relation to intensive care unit (ICU) hospitalization are presented in Table 2. Patients admitted to the ICU exhibited a significantly higher mean age by approximately 4.7 years. Serum chloride levels at admission were markedly lower in patients requiring ICU hospitalization, and notably higher D-dimer levels were observed in these patients. As an expected result, prolonged total hospital stays were evident among patients necessitating ICU care.

Using a serum chloride level threshold of 101.5 at admission, individuals surpassing this value were predicted to avoid ICU hospitalization, yielding a diagnostic success rate of 62.2% and a prediction success rate of 53.6% for identifying those likely to be ICU hospitalized.

With a D-dimer threshold of 933.5, individuals exceeding this value were anticipated to be ICU hospitalized, resulting in diagnostic success at 59.2% and predicting those unlikely to be ICU hospitalized at 53.8%.

Descriptive statistics detailing measurements in connection to mortality status are provided in Table 3. Serum chloride levels at admission were significantly lower in individuals who succumbed. Interestingly, significantly lower procalcitonin levels were evident in deceased individuals. And again, as an expected result, extended total hospital stays were observed among those who passed away.

Employing a serum chloride level threshold of 98.5 at admission, individuals surpassing this value were projected to survive, yielding a diagnostic success rate of 76.3% and predicting mortality with a success rate of 72.3%.

Utilizing a procalcitonin level threshold of 0.215, individuals exceeding this value were projected to survive, resulting in diagnostic success at 61.5% and predicting mortality with a success rate of 52.8%.

Adopting a total length of hospitalization threshold of 12.5 days, individuals surpassing this value were projected to experience mortality, yielding a diagnostic accuracy of 66.2%, while predicting survival achieved a success rate of 52.3%.

Upon segregating serum chloride measurements at the time of admission into three distinct groups and upon perusal of Table 4, it becomes evident that age, CRP, procalcitonin, and total length of stay were akin across these groups. Solely the D-dimer level exhibited statistically significant elevation in patients with hypochloremia. A comparison of these findings, with correlations accounting for direct serum chloride measurements upon admission, disclosed that solely the serum chloride levels upon admission displayed a weak yet statistically significant inverse correlation with D-dimer, while no significant associations were observed with other characteristics.

Furthermore, Table 5 presents the frequency of tocilizumab utilization, prevalence of intensive care unit hospitalizations, and mortality rate in relation to serum chloride level groupings. The frequency of tocilizumab utilization, prevalence of intensive care unit hospitalization, and mortality rate were notably higher in the hypochloremia group compared to the other two groups (normo and hyperchloremia).

## 4. Discussion

The pathogenesis of COVID-19 involves a dysregulated immune response leading to critical conditions, such as cytokine storms, ARDS, and multiorgan dysfunction [8,9]. Unraveling the complex interplay between clinical parameters and patient outcomes has become imperative. Recent research has underscored the value of novel biomarkers that can offer essential prognostic insights for system involvements, particularly for critically ill COVID-19 patients [10,11,12,13].

But identifying reliable predictors of disease severity and patient outcomes remains a pressing challenge.

Electrolytes maintain and regulate important cellular functions in our body. Electrolyte imbalances can significantly impact immune function, cellular homeostasis, and cardiovascular stability [14]. One of them is chloride, which is an important electrolyte, and is found predominantly in extracellular fluid [15]. Chloride level imbalances have been associated with poor prognosis especially in critically ill patients. Our study builds upon recent investigations that highlight the potential of hypochloremia, which was previously nominated as an indicator of disease severity and prognosis in sepsis and other specific diseases [16,17,18,19]. There are a few studies examining hypochloremia in more specific situations in COVID-19 patients [20,21]. Tezcan et al. investigated all electrolytes to find the most relevant one for a poor prognosis in COVID-19 [22]. There was also a study in the literature that did not fully reflect our study but was more similar to our study than others [23]. These significant studies emphasize the importance of monitoring serum chloride levels as a dynamic marker for assessing the progression of critical diseases including COVID-19. In light of this backdrop, our study aims to elucidate the associations between serum chloride levels upon hospital admission and critical clinical outcomes in a cohort of COVID-19 patients. By exploring the potential of hypochloremia as a prognostic biomarker, we seek to contribute to a deeper understanding of COVID-19 pathophysiology and enhance the predictive accuracy of clinical models for disease severity and patient outcomes.

Since there are no studies in the literature that can be directly compared to our work, we will highlight the key points and limitations of our study.

Our study’s gender-based analysis revealed subtle differences in serum chloride levels, albeit with limited clinical significance. Notably, tocilizumab users were characterized by higher mean ages, lower serum chloride levels, elevated D-dimer levels, and extended hospital stays. We inferred that serum chloride levels upon admission could prognosticate tocilizumab usage and, by extension, a cytokine storm, with reduced chloride levels potentially indicating the storm’s onset. A similar predictive potential of cytokine storm occurrence was also observed for D-dimer levels. Hereby, elevated D-dimer levels with hypochloremia seemed to be a significant parameter for the prediction of a bad course.

Furthermore, the integration of serum chloride levels and D-dimer thresholds proved highly informative for predicting ICU hospitalizations. Key characteristics such as age, serum chloride levels upon admission, and D-dimer levels emerged as pivotal factors among patients requiring ICU care. The potential for serum chloride levels to forecast ICU admission became evident, with low chloride levels standing out as a potential indicator. Concurrently, D-dimer levels demonstrated efficacy in predicting the need for ICU hospitalization.

Mortality prediction, which is a paramount concern, unveiled insights anchored in serum chloride levels and procalcitonin concentrations. Lower serum chloride levels reduced procalcitonin concentrations as an interesting result, and extended hospital stays were significantly associated with increased mortality rates. Lower CRP and lower procalcitonin levels detected in deceased patients could be related to immunosupression at the end stage of disease or due to the use of tocilizumab (40 of 65 deceased patients had been given tocilizumab treatment). In this regard, our study contradicts other studies that have found elevated inflammatory biomarkers in patients who died due to COVID-19 [24,25]. In our study, the fact that the majority of those who died were patients monitored in the intensive care unit is an expected finding and is consistent with other studies in the literature [26,27]. Our findings mainly underscore the capacity of serum chloride levels and procalcitonin concentrations in predicting mortality risk. Integrating these markers with other clinical parameters can refine the accuracy of prognostic models.

## 5. Conclusions

Our findings provide robust evidence supporting the value of serum chloride levels as a prognostic biomarker in critically ill COVID-19 patients. Leveraging comprehensive statistical analyses and comparisons with the relevant literature, our study strengthens the potential utility of serum chloride monitoring in refining predictive models. These insights empower healthcare practitioners with essential tools for informed clinical decision-making in the ongoing fight against COVID-19. Although our study is unique in this field, its single-center nature necessitates further large-scale studies.

## Figures and Tables

**Table 1:** Descriptive statistics of measurements contingent on tocilizumab utilization.

	Use of Tocilizumab	N	Mean	SD	Percentiles			*p* *
					25th	Median	75th	
Age	Yes	214	63.8	13.9	55	66	74	0.001
	No	285	60.6	11.03	53	61	69	
Serum chloride level at the time of admission (mmol/L)	Yes	214	100.4	7.4	95	100	107	0.001
	No	285	103.3	6.12	99	103	107	
CRP (mg/L)	Yes	214	77.9	51.6	38	74	101.15	0.204
	No	285	68.07	30.4	44.95	66	86.80	
Procalcitonin (ng/mL)	Yes	214	0.9	4.48	0.12	0.21	0.83	0.911
	No	285	0.56	0.79	0.12	0.23	0.82	
D-dimer (µg/L)	Yes	214	3557.3	4665.2	786.75	2098	4390.50	0.001
	No	285	1313.6	1577.05	498	812	1313	
Total length of hospital stay (days)	Yes	214	15.4	5.28	11	14	19	0.001
	No	285	13.02	4.84	10	12	16	

*: Mann–Whitney U test.

**Table 2:** Descriptive statistics pertaining to measurements in relation to intensive care unit hospitalization.

	Hospitalization in Intensive Care Unit	N	Mean	SD	Percentiles			*p* *
					25th	Median	75th	
Age	Yes	250	64.3	12.5	57	66	73	0.001
	No	249	59.6	11.8	52	60	69	
Serum chloride level at the time of admission (mmol/L)	Yes	250	101.2	7.25	96	101	106	0.001
	No	249	102.9	6.31	99	103	107	
CRP (mg/L)	Yes	250	74.5	47.08	41.70	71.85	97.45	0.683
	No	249	70.09	34.2	43.50	67.30	90.20	
Procalcitonin (ng/mL)	Yes	250	0.86	4.15	0.12	0.28	0.88	0.129
	No	249	0.56	0.83	0.12	0.18	0.75	
D-dimer (µg/L)	Yes	250	2771.8	3916.7	678.7	115	3054.75	0.001
	No	249	1777.9	2849.9	560	895	1883.50	
Total length of hospital stay (days)	Yes	250	15.5	5.49	11	15.50	19	0.001
	No	249	12.5	4.33	10	11	16	

*: Mann–Whitney U test.

**Table 3:** Descriptive statistics detailing measurements in connection to mortality status.

	Death	N	Mean	SD	Percentiles			*p* *
					25th	Median	75th	
Age	Yes	65	63.1	11.9	56.50	63	72	0.478
	No	434	61.8	12.5	53	64	71	
Serum chloride level at the time of admission (mmol/L)	Yes	65	96.5	7.35	90	96	101	0.001
	No	434	102.9	6.37	99	103	107	
CRP (mg/L)	Yes	65	68.2	41.2	35.35	66.50	92.25	0.346
	No	434	72.9	41.2	43.95	71.85	92.30	
Procalcitonin (ng/mL)	Yes	65	0.40	0.62	0.12	0.12	0.60	0.027
	No	434	0.76	3.20	0.12	0.25	0.86	
D-dimer (µg/L)	Yes	65	2646.2	3212.7	660.50	1868	3224	0.079
	No	434	2220.4	3494.1	579	984.50	2356	
Total length of hospital stay (days)	Yes	65	15.6	5.58	11	16	18	0.011
	No	434	13.8	5.06	10	12	17	

*: Mann–Whitney U test.

**Table 4:** Descriptive statistical analyses of other measurements in relation to serum chloride level groups.

	Serum Chloride Level Group	N	Mean	SD	Percentiles			*p* *
					25th	Median	75th	
Age	Hypochloremia	123	61.8	14.07	52	64	72	0.907
	Normal	283	62.2	11.2	55	63	70	
	Hyperchloremia	93	61.3	13.5	53	64	73	
CRP (mg/L)	Hypochloremia	123	70.7	42.6	37.50	66.90	92.90	0.276
	Normal	283	74.6	43.3	45	73	96.80	
	Hyperchloremia	93	67.09	31.1	43.40	66.40	83.45	
Procalcitonin (ng/mL)	Hypochloremia	123	0.47	0.62	0.12	0.19	0.66	0.397
	Normal	283	0.85	3.92	0.12	0.24	0.88	
	Hyperchloremia	93	0.60	0.90	0.12	0.30	0.84	
D-dimer (µg/L)	Hypochloremia	123	2747.49	3209.3	703	1439	3256	0.049
	Normal	283	2182.86	3821.7	571	913	2108	
	Hyperchloremia	93	1935.24	2426.09	630.50	985	2330.50	
Total length of hospital stay (days)	Hypochloremia	123	14.30	4.95	10	13	18	0.567
	Normal	283	14.04	5.24	10	13	17	
	Hyperchloremia	93	13.7	5.23	10	12	17.50	

*: Kruskal–Wallis test.

**Table 5:** Frequency of tocilizumab utilization, prevalence of intensive care unit hospitalizations, and mortality rate in relation to serum chloride level groupings.

		Hypochloremia (n = 123)		Normal (n = 283)		Hyperchloremia (n = 93)		*p* *
		n	%	n	%	n	%	
Tocilizumab use	Yes	80	65.0 ^a^	96	33.9 ^bc^	38	40.9 ^bc^	0.001
	No	43	35.0 ^a^	187	66.1 ^bc^	55	59.1 ^bc^	
Hospitalization in intensive care unit	Yes	77	62.6 ^a^	131	46.3 ^bc^	42	45.2 ^bc^	0.006
	No	46	37.4 ^a^	152	53.7 ^b^	51	54.8 ^bc^	
Death	Yes	45 ^a^	36.6 ^a^	14	4.9 ^bc^	6	6.5 ^bc^	0.001
	No	78	63.4 ^a^	269	95.1 ^bc^	87	93.5 ^bc^	

*: Pearson chi-square test. ^a, b, c^: If two parameters have different letters (such as ‘a’ and ‘b’), it means they are significantly different. If they share the same letter or have a common letter (such as ‘a’ and ‘a’ or ‘a’ and ‘ab’), they are not significantly different.

## Data Availability

Data are contained within the Supplementary Materials.

## Supplementary Materials

The following supporting information can be downloaded at: https://zenodo.org/records/12594056 (accessed on 27 August 2024).
